# New 3D-Culture Approaches to Study Interactions of Bone Marrow Adipocytes with Metastatic Prostate Cancer Cells

**DOI:** 10.3389/fendo.2016.00084

**Published:** 2016-07-06

**Authors:** Mackenzie Katheryn Herroon, Jonathan Driscoll Diedrich, Izabela Podgorski

**Affiliations:** ^1^Department of Pharmacology, Wayne State University School of Medicine, Detroit, MI, USA; ^2^Karmanos Cancer Institute, Wayne State University School of Medicine, Detroit, MI, USA

**Keywords:** bone marrow adipocytes, bone metastasis, prostate cancer, 3D culture, 3D invasion, *in vitro* models

## Abstract

Adipocytes are a major component of the bone marrow that can critically affect metastatic progression in bone. Understanding how the marrow fat cells influence growth, behavior, and survival of tumor cells requires utilization of *in vitro* cell systems that can closely mimic the physiological microenvironment. Herein, we present two new three-dimensional (3D) culture approaches to study adipocyte–tumor cell interactions *in vitro*. The first is a transwell-based system composed of the marrow-derived adipocytes in 3D collagen I gels and reconstituted basement membrane-overlayed prostate tumor cell spheroids. Tumor cells cultured under these 3D conditions are continuously exposed to adipocyte-derived factors, and their response can be evaluated by morphological and immunohistochemical analyses. We show via immunofluorescence analysis of metabolism-associated proteins that under 3D conditions tumor cells have significantly different metabolic response to adipocytes than tumor cells grown in 2D culture. We also demonstrate that this model allows for incorporation of other cell types, such as bone marrow macrophages, and utilization of dye-quenched collagen substrates for examination of proteolysis-driven responses to adipocyte- and macrophage-derived factors. Our second 3D culture system is designed to study tumor cell invasion toward the adipocytes and the consequent interaction between the two cell types. In this model, marrow adipocytes are separated from the fluorescently labeled tumor cells by a layer of collagen I. At designated time points, adipocytes are stained with BODIPY and confocal *z*-stacks are taken through the depth of the entire culture to determine the distance traveled between the two cell types over time. We demonstrate that this system can be utilized to study effects of candidate factors on tumor invasion toward the adipocytes. We also show that immunohistochemical analyses can be performed to evaluate the impact of direct interaction of prostate tumor cells with adipocytes. Our models underline the importance of using the appropriate culture conditions to mimic physiological interactions between marrow adipocytes and metastatic tumor cells. These systems have a potential to be utilized for analyses of various factors that may be regulated by the adipocytes in bone. Their application likely extends beyond metastatic prostate cancer to other tumors that colonize the bone marrow microenvironment.

## Introduction

Adipocytes constitute a significant portion of adult bone marrow and their number increases with age, obesity, and metabolic dysfunction (1–3). Growing evidence positively links the abundance of fat cells in the marrow with metastatic progression. Adipocyte-rich bone marrow appears to contribute to skeletal colonization and growth in a number of secondary cancers, including prostate (4, 5), breast (6), multiple myeloma (7, 8), and melanoma tumors (9). It is believed that adipocytes enhance the fertility of the bone metastatic niche by serving as a source of growth factors, chemokines, and lipid mediators (10, 11). Specifically, they have been shown to (1) upregulate lipid transporters and drive lipid uptake by tumor cells (5), (2) promote osteoclast differentiation and maturation (4, 9), and (3) induce authophagy-driven tumor cell survival, all processes that ultimately allow the metastatic cancers to thrive in the bone marrow niche (8). Despite these emerging data clearly pointing to marrow fat cells as one of the critical determinants of tumor cell fate in bone, their functional contribution to the growth and aggressiveness of metastatic tumors in bone is not well understood. Studies investigating the interactions between the tumor cells and adipocytes in the bone marrow have been limited and thorough mechanistic evaluations on how fat cells affect the phenotype, metabolism, and function of the surrounding cells in the metastatic niche are lacking.

The majority of the studies examining adipocyte–tumor cell interactions to date have utilized pre-adipocyte cell lines or adipocytes derived from visceral or breast adipose tissues (12–16) depots, which are known to be distinctively different from bone marrow fat (17). There have only been a handful of studies, including our own, that have examined the interactions of bone marrow mesenchymal cell-derived or primary bone adipocytes with metastatic tumor cells (4, 5, 7–9). Although all of these investigations resulted in important findings linking marrow adipocytes with metastatic progression, the caveat is that they have all been performed using two-dimensional (2D) culture approaches. It is becoming increasingly recognized that 2D layer cultures, although convenient and reasonably inexpensive, do not adequately mimic the limited diffusion-driven access to nutrients, growth factors, and signaling molecules in the tumor microenvironment (18). Under physiological conditions, exposure of solid tumors to microenvironmental factors, such as oxygen, nutrients, stress, and therapeutic treatments, is heterogeneous and regulated by their three-dimensional (3D) spatial conformation (19). The importance of employing 3D models to model tumor architecture has proven critical to understanding the mechanisms behind tumor phenotype, behavior, and response to therapy (19–22). Emphasis has also grown on considering the contribution of host cells in the tumor microenvironment to cancer progression, and various *in vitro* models that focus on stromal–epithelial interactions and immune cell involvement have emerged (21, 23–27).

Three-dimensional, multi-cellular cell culture models have become well-accepted tools for dissecting complex molecular mechanisms of tumor progression that may not be possible to dissect *in vivo*. There have also been many advancements in the development of 3D culture systems that mimic specific tumor niches, including very complex and dynamic microenvironments such as bone (28). Models intended to interrogate the mechanisms of skeletal metastases range from culture of tumor cells and specific bone-derived cells on biologically derived or synthetic matrices, through the use of patient-derived xenografts and direct culture of tumor cells with bone explants (21, 28). However, aside from one recently reported *in vitro* system designed to evaluate bone marrow adipose colonization by breast cancer cells (6), there have been no *in vitro* 3D models that consider involvement of marrow adipocytes.

Here, we describe new *in vitro* approaches designed to study the interaction of prostate cancer cells with bone marrow-derived adipocytes. Our methods employ murine bone marrow mesenchymal cells differentiated into adipocytes in 3D collagen I gel and grown in a Transwell system with 3D-cultures of prostate carcinoma cells. We show that in this system, which allows continuous exchange of factors between the two cell types, adipocytes promote 3D growth of tumor spheroids. We also demonstrate that the cell culture approaches we are employing in this model allow for easy manipulation and are suitable for imunocytochemical analyses. We show examples of immunofluorescence analyses of metabolism-associated factors, such as carbonic anhydrase 9 (CA9) and hexokinase 2 (HK2) that reveal distinctively different expression profiles between 2D and 3D cultures exposed to adipocytes. We also demonstrate the suitability of our model to study proteolysis by live prostate carcinoma cells and potentially other components of bone marrow microenvironment, such as bone marrow macrophages. Finally, we also describe a design of a 3D invasion assay that allows direct monitoring of the attraction of prostate tumor cells to marrow adipocytes and can be utilized to evaluate potential inhibitors that target this interaction. Our models provide new approaches to dissect the functional role of marrow-derived adipocytes in tumor cell growth and aggressiveness.

## Materials and Methods

### Materials

Dulbecco’s modified Eagle’s medium (DMEM), minimum essential medium (MEMα), and other chemicals, unless otherwise stated, were obtained from Sigma (St. Louis, MO, USA). HyClone fetal bovine serum (FBS) was from ThermoFisher (Pittsburg, PA, USA). Trypsin–EDTA, Alexa Fluor 488-conjugated goat anti-rat and anti-rabbit IgG, MitoTracker Deep Red FM, CellTracker Orange (CTO), DQ collagen type IV, BODIPY (493/503), Hoechst Dye, and Gentamicin (G418) were from Invitrogen (Carlsbad, CA, USA). StemXVivo Adipogenic Supplement was from R&D Systems (Minneapolis, MN, USA). Rosiglitazone was from Cayman Chemical Company (Ann Arbor, MI, USA). PureCol collagen type I was from Advanced Biomatrix (San Diego, CA, USA). Cultrex™ (rBM; reduced growth factor) was from Trevigen (Gaithersburg, MD, USA). Rat monoclonal F4/80 was from Abcam (Cambridge, MA, USA). Rabbit monoclonal carbonic anhydrase 9 (CA9) and hexokinase II (HK2) were from Cell Signaling Technology (Danvers, MA, USA). Transwell systems (Costar™ Transwell™ Permeable Supports with 0.4-μm pore size) were from Corning (Corning, NY, USA). FABP4 inhibitor (BMS309403) was from Calbiochem (San Diego, CA, USA). Atglistatin was from Axon Medchem (Groningen, Netherlands).

### Cell Lines

PC3, an androgen independent cell line derived from a bone metastasis of a high-grade prostate adenocarcinoma, was purchased from American Type Culture Collection (ATCC; Manassas, VA, USA). The PC3-DsRed cell line was established by stable transfection with pDsRed2-N1 vector (Clontech Laboratories, Palo Alto, CA, USA), containing the neomycin-resistant gene. Transfection was performed using Lipofectamine 2000 and pooled populations of stable cells were selected, expanded, and maintained in medium supplemented with 400 mg/ml of G418 (29). L929 cells (source of M-CSF for macrophages, purchased from ATCC) were cultured until confluent and conditioned medium was collected, centrifuged, and stored at −80°C until ready for use. PC3 and L929 cells were cultured in DMEM supplemented with 10% FBS, 10 mM HEPES, and 100 U/ml penicillin–streptomycin. All cells were maintained in a 37°C humidified incubator ventilated with 5% CO_2_.

### Isolation and Preparation of Primary Murine Bone Marrow Adipocytes and Macrophages

Collection of murine cells was performed in accordance with the protocol approved by the institutional Animal Investigational Committee of Wayne State University and NIH guidelines (Protocol # 15-12-025; IP, PI). Primary mouse bone marrow stromal cells (mBMSC) were isolated from femurs and tibiae of 6- to 8-week-old FVB/N mice and induced to become bone marrow adipocytes according to our previously published protocols (5). Specifically, bone marrow from each tibia and femur was flushed with of DMEM containing 20% FBS (4 ml/bone) using a 26-gauge needle. Marrow suspension was mixed and broken apart using a 20-gauge needle and seeded into a 6-well cell culture plate (~3 ml/well). After 24 h, non-adherent cells were removed by replacing the medium. Cells were cultured to confluency by changing the medium every 2–3 days and then expanded to larger dishes as needed. For adipogenic differentiation, mBMSCs (~600,000/well) were mixed with bovine collagen I and seeded in six-well plates (500 μl/well) for Transwell coculture or 60-mm dishes (500 μl, ~1,300,000/dish) for 3D invasion assays. Approximately 48–72 h later, upon reaching confluency, cells were treated with adipogenic cocktail (30% StemXVivo Adipogenic Supplement, 1 μM insulin, 2 μM Rosiglitazone; DMEM and 10% FBS) for 8–10 days (5).

For the preparation of bone marrow macrophages (BMMs), bone marrow was flushed from femurs and tibiae of 10- to 12-week-old FVB/N male mice with BMM growth medium (MEMα containing 20% FBS and 30% L929-conditioned media as the source of M-CSF; 4 ml/bone) using a 26-gauge needle (30). The cell suspension was mixed with an additional 20 ml of BMM medium using a 20-gauge needle and plated onto three 100-mm Petri dishes (12 ml/dish). Cells were cultured for 4–5 days in a 37°C humidified incubator ventilated with 5% CO_2_ to obtain differentiated BMMs.

### 2D Tumor Cell-Adipocyte Coculture Using a Transwell System

Bone marrow adipocytes were differentiated in six-well plates as described above. On the day of the Transwell setup, tumor cells were plated on acid-washed glass coverslips (12 mm in diameter) coated with reconstituted basement membrane (rBM; Cultrex™; Trevigen). Briefly, 100 μl of 1 mg/ml rBM was added per coverslip and allowed to polymerize for 45 min at RT, then the excess was removed via suction, and the coverslips were allowed to dry for 15 min at RT before cells were plated. PC3 cells (30,000–50,000) were plated in 500 μl of normal growth medium and allowed to settle for a minimum of 4 h at 37°C. Adipocytes were prepared for Transwell coculture by washing 3× with PBS and adding 2 ml of DMEM supplemented with 5% FBS. Transwell membranes were placed above the adipocyte culture according to manufacturer’s instructions and coverslips with attached tumor cells (2× coverslips per transwell) were placed on each membrane with medium gently added on top (2 ml). The control coverslips were cultured separately in DMEM supplemented with 5% FBS. All cells were allowed to grow for 48 h at 37°C.

### 3D Tumor Cell-Adipocyte Coculture Using a Transwell System

**Figure 1:**
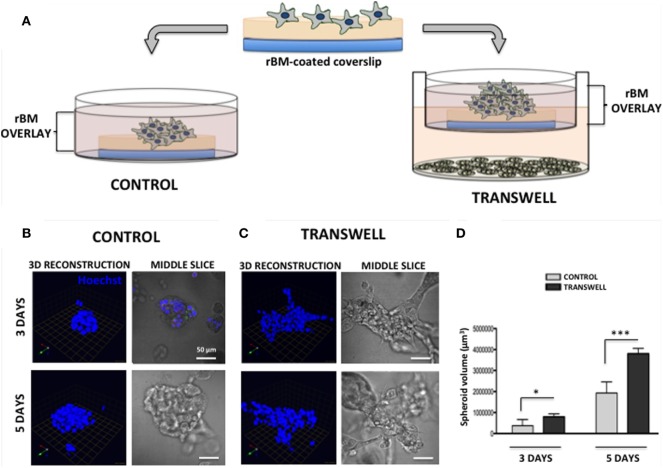
**Three-dimensional (3D) culture of prostate tumor spheroids in the absence or presence of bone marrow-derived adipocytes**. **(A)** Schematic representation of 3D culture of tumor cells alone plated on reconstituted basement membrane (rBM)-coated coverslips with rBM overlay of 2% rBM and cultured alone (left) or in transwell with bone marrow adipocytes (right). **(B)** 3D reconstruction and DIC (differential interference contrast) images of the middle slice depicting morphology of the spheroids of PC3 cells grown for 3 (top) and 5 (bottom) days in control conditions or **(C)** in transwell coculture with adipocytes. 40× images; bar, 50 μm; nuclei are labeled with Hoechst dye (blue); green arrow (*X*), red arrow (*Y*), and blue arrow (*Z*) indicate orientation of the spheroid in 3D space. **(D)** Quantification of spheroid volume using Volocity software; data are shown in cubic micrometers and represent the mean (±SD) of three independent experiments with at least three independent spheroids measured/experiment; *p < 0.05 and ***p < 0.001 are considered statistically significant.

Adipocytes were prepared as described above for the 2D system. The experimental approach to prepare 3D cultures of prostate carcinoma cells has been adapted from the original protocols for breast cancer progression models developed by the Brugge laboratory (31). Non-diluted rBM (15.35 mg/ml) was used for the preparation of all cultures. In brief, acid-washed coverslips were placed in 35 mm dishes, and 45 μl of rBM was carefully added on top of each coverslip, making a continuous surface without going over the edge of the coverslip. The dishes were placed in 37°C for 15 min to polymerize, then 60 μl of PC3 cell suspension was placed on top of each rBM-coated coverslip (30,000–35,000 cells/coverslip). The dishes were placed in a 37°C incubator for 45 min–1 h to allow the cells to settle and attach to the matrix. After cells were attached, 2–3 ml of media (5% FBS, 2% rBM overlay) was added to the 35-mm dish control coverslips. For 3D Transwell cultures, one 3D coverslip (covering only ~24% of the membrane and thus allowing free flow of factors and nutrients) was placed on each Transwell membrane positioned above the differentiated adipocyte culture, and 2 ml of medium was gently added (Figure 1). Cells were allowed to grow for 48–120 h depending on experimental design.

### Immunofluorescence Analyses

**Figure 2:**
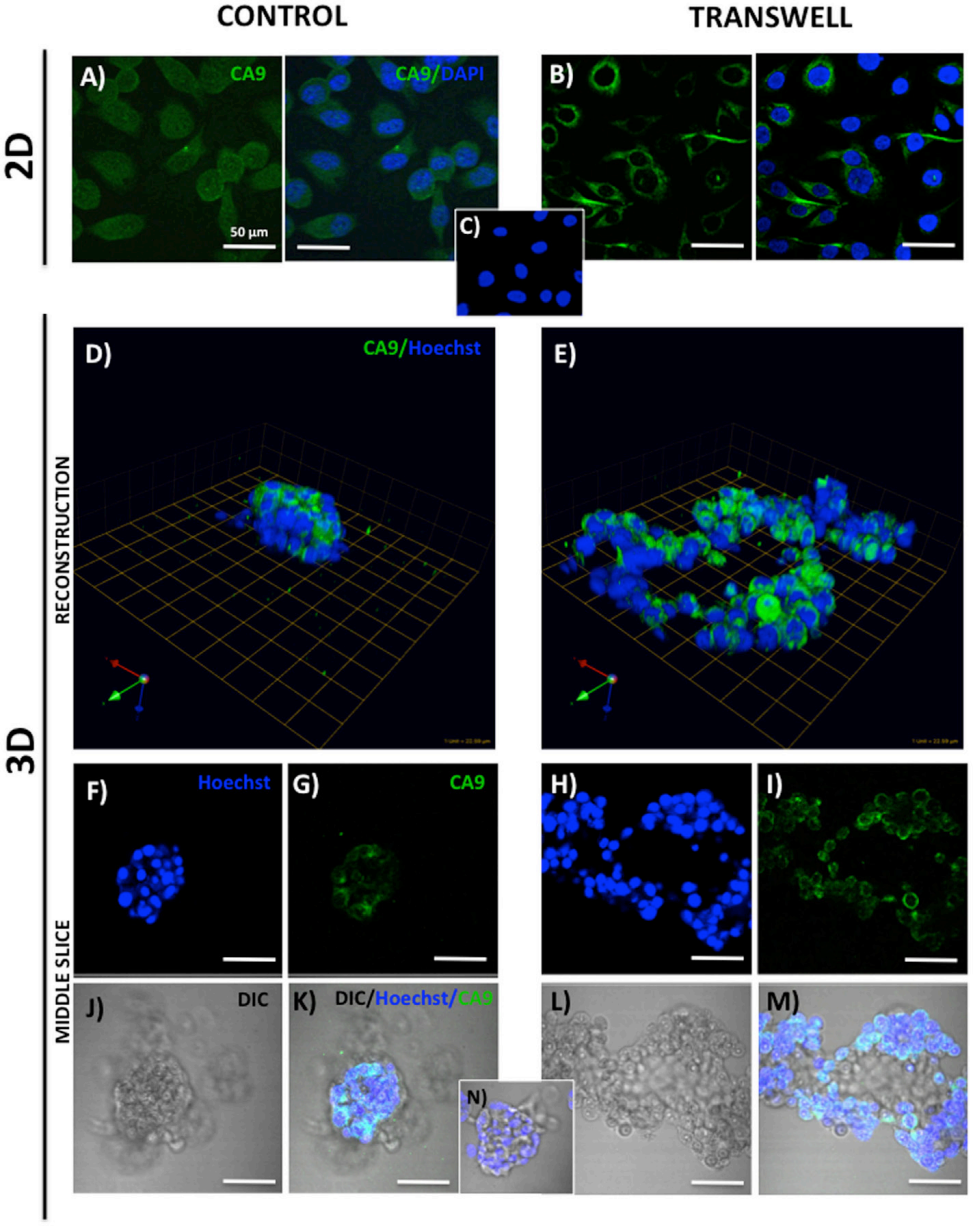
**Expression of carbonic anhydrase 9 (CA9) in 2D and 3D cultures of PC3 cells grown alone or in transwell coculture with adipocytes**. Immunofluorescence analysis of CA9 expression (green) in monolayer PC3 cultures grown alone **(A)** or in transwell with adipocytes **(B)**. **(C)** 2D no primary CA9 antibody control; DAPI was used as nuclear marker (Blue). 3D reconstruction of CA9 expression in tumor spheroids grown alone **(D)** or in transwell with adipocytes **(E)**; green arrow (*X*), red arrow (*Y*), and blue arrow (*Z*) indicate orientation of the spheroid in 3D space. Middle slice through *z*-stack showing CA9 expression in spheroids cultured alone **(F,G,J,K)** or in transwell with adipocytes **(H,I,L,M)**. Hoechst dye was used for labeling nuclei [**(F,H)**; blue], CA9 [**(G,I)**; green], DIC [**(J,L)**; differential interference contrast], and merged images **(K,M)**. **(N)** 3D culture; no CA9 primary antibody control; 40× images; bar, 50 μm.

**Figure 3:**
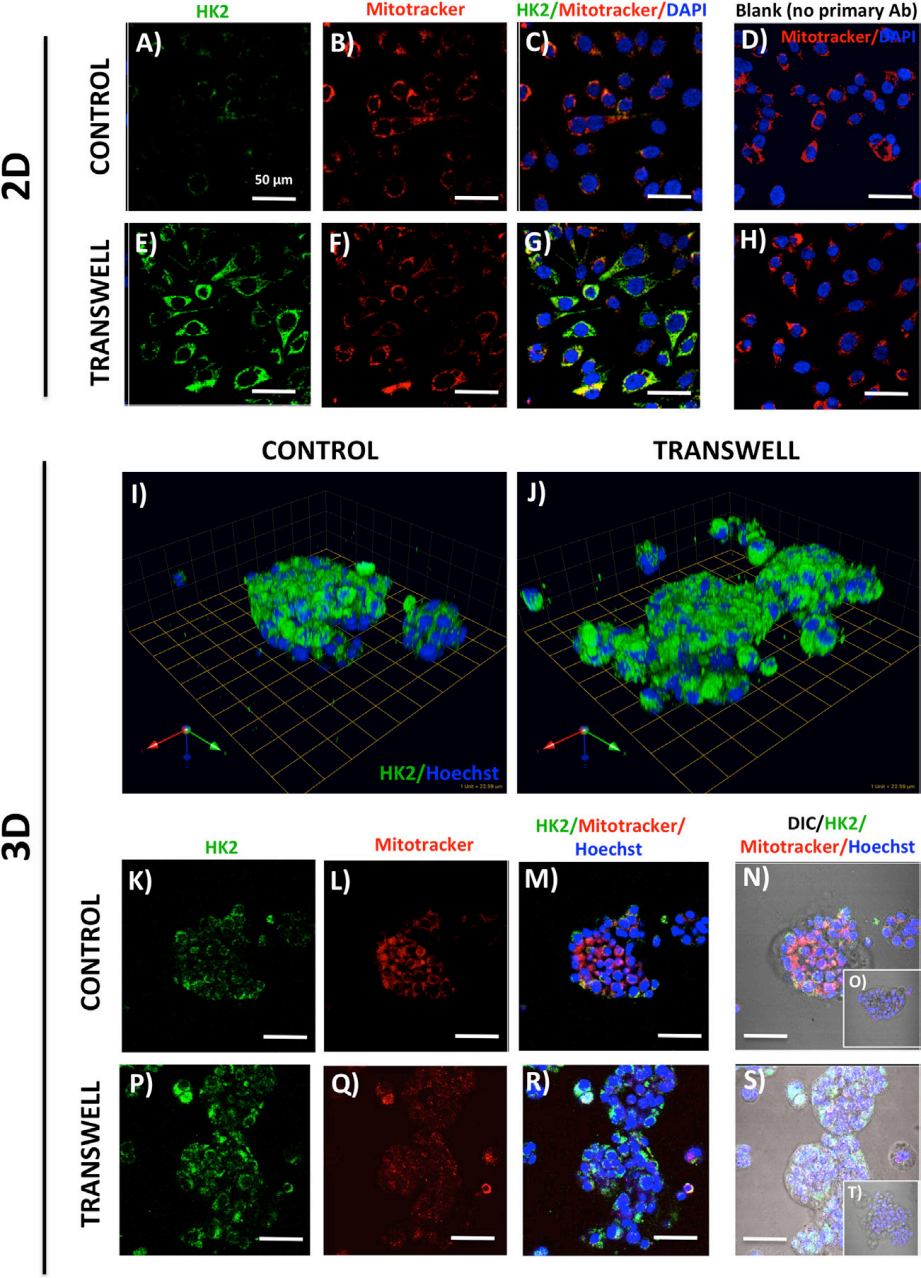
**Hexokinase 2 (HK2) expression and in 2D and 3D cultures of PC3 cells grown alone or in transwell coculture with adipocytes**. Expression of HK2 [**(A)**, green] and MitoTracker [**(B)**, red] merged **(C)** in PC3 cells grown alone **(A–C)** or in transwell **(E–G)** in 2D coculture. **(D,H)** No HK2 primary antibody controls; DAPI was used as nuclear marker (blue). **(I,J)** 3D reconstruction of CA9 expression in tumor spheroids grown alone **(I)** or in transwell with adipocytes **(J)**; green arrow (*X*), red arrow (*Y*), and blue arrow (*Z*) indicate orientation of the spheroid in 3D space. HK2 and MitoTracker fluorescence to show HK2 localization to the mitochondria in PC3 cells cultured alone **(K–N)** or in Transwell with adipocytes **(P–S)**. **(O,T)** No HK2 primary antibody controls; 40× images; bar, 50 μm.

For the analysis of the expression and localization of carbonic anhydrase 9 (CA9) (Figure 2), control and Transwell coverslips from 2D and 3D cultures were washed with PBS, fixed with cold methanol, and incubated with rabbit monoclonal anti-CA9 antibody (1:50) at 4°C overnight. To label the mitochondria, coverslips were washed with PBS, and incubated with 200 nM MitoTracker Deep Red for 30–45 min at 37°C prior to fixation. For immunodetection of hexokinase 2 (HK2) (Figure 3), cells were washed with PBS, fixed with cold methanol, and stained with rabbit monoclonal anti-HK2 antibody (1:100) at 4°C overnight. Alexa Fluor 488-conjugated goat anti-rabbit IgG (1:1000) was used as a secondary antibody for both CA9 and HK2 immunostaining. For 2D cultures, DAPI was used as a nuclear stain, and coverslips were mounted using Vectashield mounting medium (Vector Laboratories) before imaging with Zeiss LSM 510 META NLO confocal microscope using 40× oil immersion lens. Coverslips from 3D cultures were left in PBS in 35-mm dishes, Hoechst Dye (1:1000) was added for labeling of nuclei, and imaging was performed at an extended depth of focus with a Zeiss LSM 510 META NLO confocal microscope using a 40× dipping lens.

### Imaging the Infiltration of Bone Marrow Macrophage into 3D Tumor Spheroids

**Figure 4:**
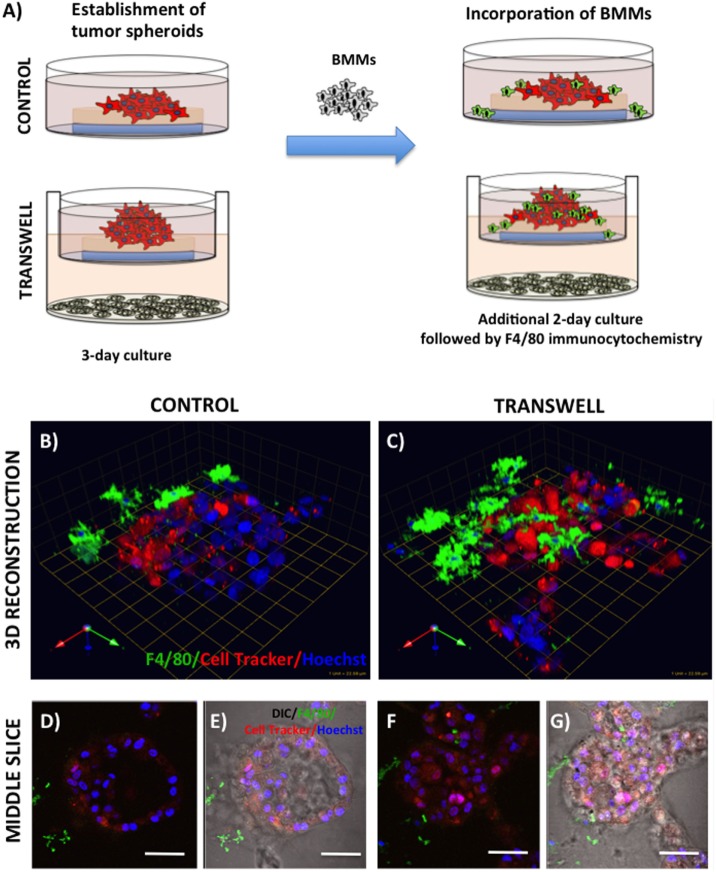
**Infiltration of bone marrow macrophages (BMMs) into 3D cultures of PC3 cells exposed to adipocytes**. **(A)** Schematic representation of experimental design. 3D cultures were grown alone or in transwell coculture with adipocytes for three days prior to addition of bone marrow macrophages and culture for an additional 2 days. **(B,C)** 3D reconstruction of tumor spheroids infiltrated with BMMs in the absence **(B)** or presence of adipocytes **(C)**; F4/80: macrophage marker (green); CellTracker Orange: PC3 cell label (red); Hoechst: nuclear label (blue); green arrow (*X*), red arrow (*Y*), and blue arrow (*Z*) indicate orientation of the spheroid in 3D space. Middle slice through *z*-stack showing F4/80, cell tracker, and Hoechst staining of PC3 spheroids with or without BMMs grown in control conditions **(D,E)** or in transwell with adipocytes **(F,G)**; 40× images; bar, 50 μm

Primary BMMs were differentiated from murine bone marrow cells, as described above. To examine their ability to infiltrate the pre-formed tumor spheroids in the absence or presence of adipocytes, the 3D tumor-adipocyte Transwell cocultures were set up and cultured for 3 days, as described above. To distinguish tumor cells from macrophages, PC3 cells were pre-labeled with CTO (1:1000 in serum-free media for 1 h at 37°C) prior to plating on rBM-covered coverslips. After 3-day culture, medium with rBM overlay was removed from the Transwell or control dishes, 60 μl of cell suspension containing 150,000 BMMs was plated on top of each coverslip and allowed to settle for 45min-1 h at 37°C, then new medium (containing 2% rBM overlay) was reapplied. Cells were allowed to grow for additional 2 days (Figure 4) before cultures were fixed in 3.7% formaldehyde and immunostained for murine macrophage marker F4/80 using rat anti-mouse F4/80 antibody (1:50). Alexa Fluor 488 conjugated goat anti-rat IgG (1:1000) was used as a secondary antibody. Hoechst Dye (1:1000) was added for labeling of nuclei, and imaging was performed at an extended depth of focus with Zeiss LSM 510 META NLO confocal microscope using a 40× dipping lens.

### Imaging Proteolysis by Live Prostate Tumor Cells in the Absence or Presence of Infiltrating BMMs

**Figure 5:**
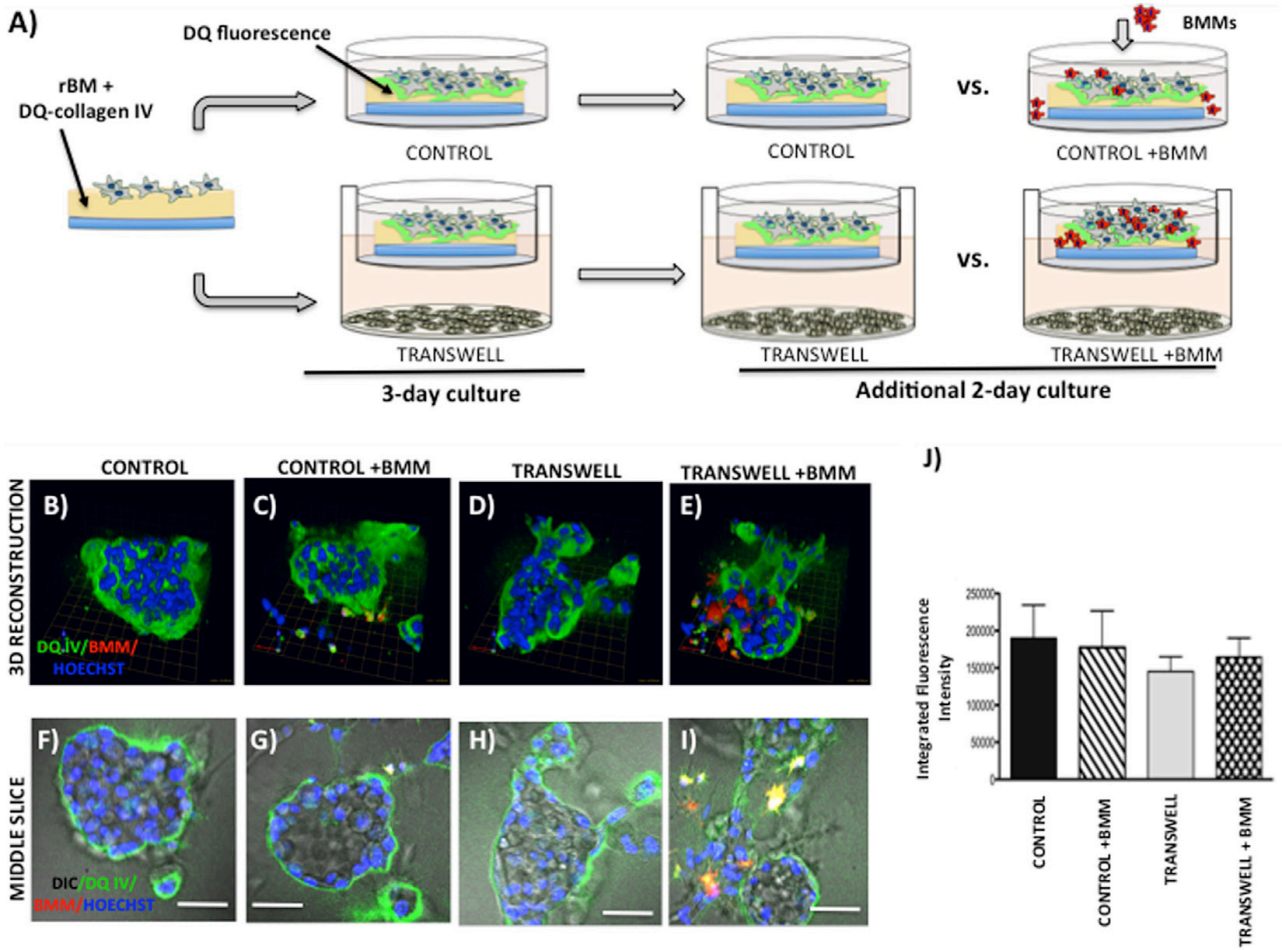
**Live proteolysis by 3D prostate tumor cell cultures in the absence or presence of infiltrating macrophages**. **(A)** Schematic representation of experimental design. 3D cultures were seeded on rBM containing DQ-Collagen IV substrate and grown alone or in transwell coculture with adipocytes for 3 days prior to addition of bone marrow macrophages and culture for an additional 2 days. **(B–E)** 3D reconstruction of DQIV proteolysis by tumor spheroids cultured alone **(B)** or infiltrated with BMMs **(C)** in the absence or presence of adipocytes **(D,E)**; DQ-Collagen IV cleavage products (green), CellTracker Orange: PC3 cell label (red); Hoechst: nuclear label (blue); green arrow (*X*), red arrow (*Y*), and blue arrow (*Z*) indicate orientation of the spheroid in 3D space. **(F–I)** Middle slice through *z*-stack showing DQIV fluorescence, CellTracker Orange, and Hoechst staining of PC3 spheroids with or without BMMs grown in control conditions **(F,G)** or in transwell with adipocytes **(H,I)**; 40× images; bar, 50 μm. **(J)** Quantification of DQ-Collagen IV proteolysis shown as fluorescence intensity per cell in the entire volume; nuclei were stained with Hoechst 33342 (*blue*) at the time of imaging and counted. Data are shown as average (±SD) of three independent experiments with at least three independent spheroids measured/experiment.

Cleavage of DQ-collagen IV substrate by live PC3 cells grown in a control or Transwell coculture with adipocytes in the presence or absence of BMMs was assayed in real time and quantified based on published protocols (32–34). Briefly, single cell suspensions of tumor cells (30,000–35,000) were plated on top of coverslips coated with rBM containing DQ-collagen IV (1:30) and overlayed with 2% rBM. Cells were grown alone or in Transwell coculture with adipocytes for 3 days prior to addition of pre-labeled BMMs (Figure 5). After an additional 2-day culture, Hoechst dye was added as nuclear marker and DQ-IV proteolysis under all culture conditions was imaged live at an extended depth of focus with Zeiss LSM 510 META NLO confocal microscope using a 40× dipping lens.

### Coculture of Bone Marrow Macrophages with Prostate Tumor Cells in a 3D Transwell System

**Figure 6:**
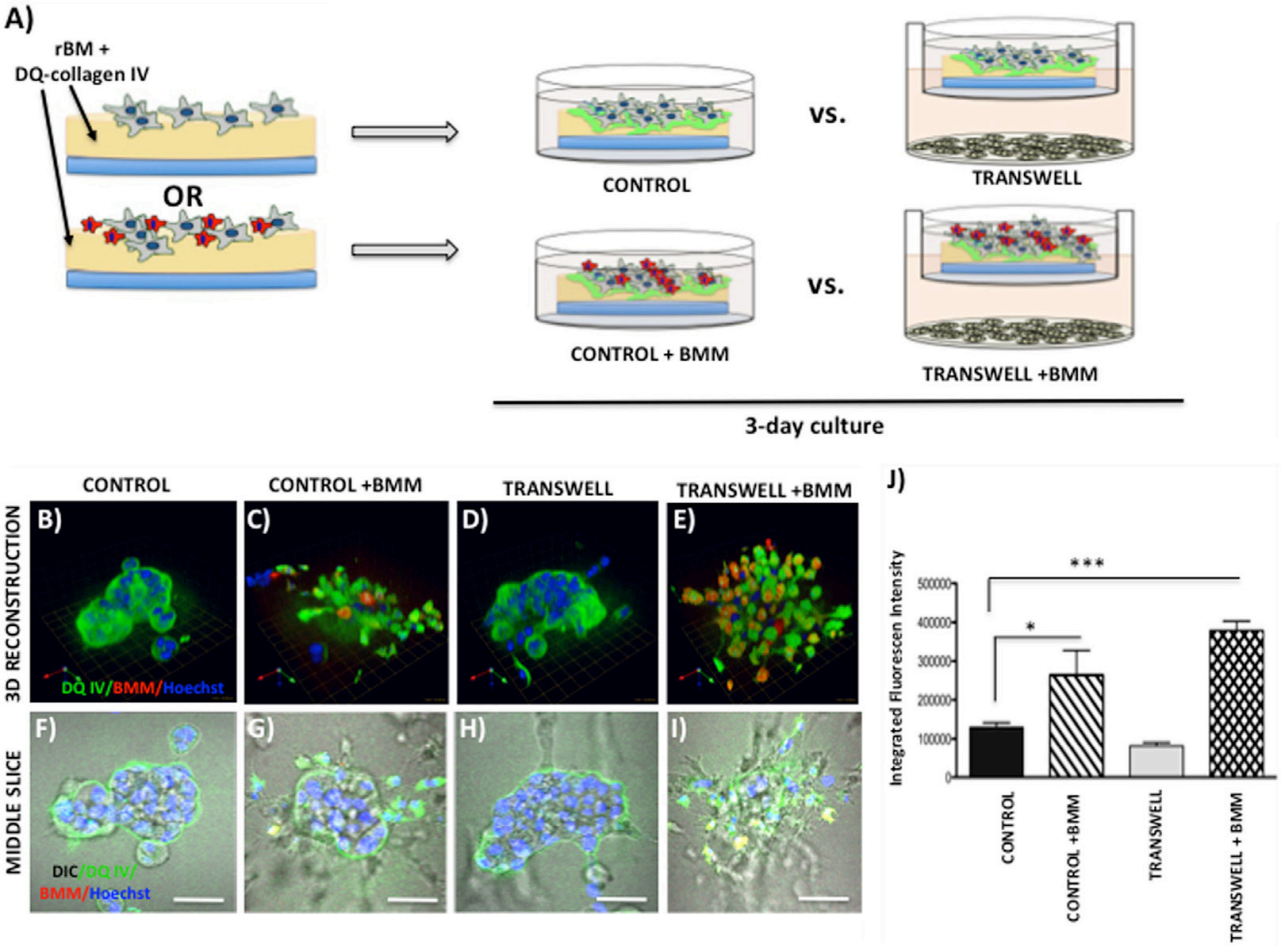
**Contribution of bone marrow macrophages to proteolysis of DQ-Collagen IV by tumor cells exposed to bone marrow adipocytes**. **(A)** Schematic representation of experimental design. Tumor cells were mixed with BMMs, and 3D cultures were seeded on rBM containing DQ-Collagen IV substrate and grown alone or in transwell coculture with adipocytes for 3 days. **(B–E)** 3D reconstruction of DQIV proteolysis by tumor spheroids seeded alone **(B)** or with BMMs **(C)** in the absence or presence of adipocytes **(D,E)**; DQ-Collagen IV cleavage products (green), CellTracker Orange: PC3 cell label (red); Hoechst: nuclear label (blue); green arrow (*X*), red arrow (*Y*), and blue arrow (*Z*) indicate orientation of the spheroid in 3D space. **(F–I)** Middle slice through *z*-stack showing DQIV fluorescence, CellTracker Orange, and Hoechst staining of PC3 spheroids with or without BMMs grown in control conditions **(F,G)** or in transwell with adipocytes **(H,I)**; 40× images; bar, 50 μm. **(J)** Quantification of DQ-Collagen IV proteolysis, quantified in each 3D reconstructed spheroid using Volocity Software, is shown as fluorescence intensity per cell in the entire volume; nuclei were stained with Hoechst 33342 (*blue*) at the time of imaging and counted. Data are shown as average (±SD) of three independent experiments with at least three independent spheroids measured/experiment. *p < 0.05 and ***p < 0.001 are considered statistically significant.

To examine the effects of BMMs on 3D growth and proteolysis by prostate tumor cells cocultured with marrow adipocytes, we pre-labeled differentiated BMMs by incubating them with a 1:1000 dilution of with CellTracker Orange (CTO) in serum-free medium for 1 h at 37°C. Labeled BMMs were then washed 3× with PBS and maintained in normal BMM medium overnight until use. On the day of 3D Transwell setup, adipocytes and prostate tumor cells were prepared as described above. Coverslips were coated with rBM and 75,000 BMMs mixed with 30,000 PC3 cells were plated together on top of a coated coverslip, as described above. As before, one 3D coverslip was placed on each Transwell membrane positioned above differentiated adipocyte culture, and 2 ml of medium was gently added (Figure 6). Cells were allowed to grow for 3 days, and DQ-IV proteolysis was imaged live at an extended depth of focus with a Zeiss LSM 510 META NLO confocal microscope using a 40× dipping lens.

### 3D Invasion Assays

**Figure 7:**
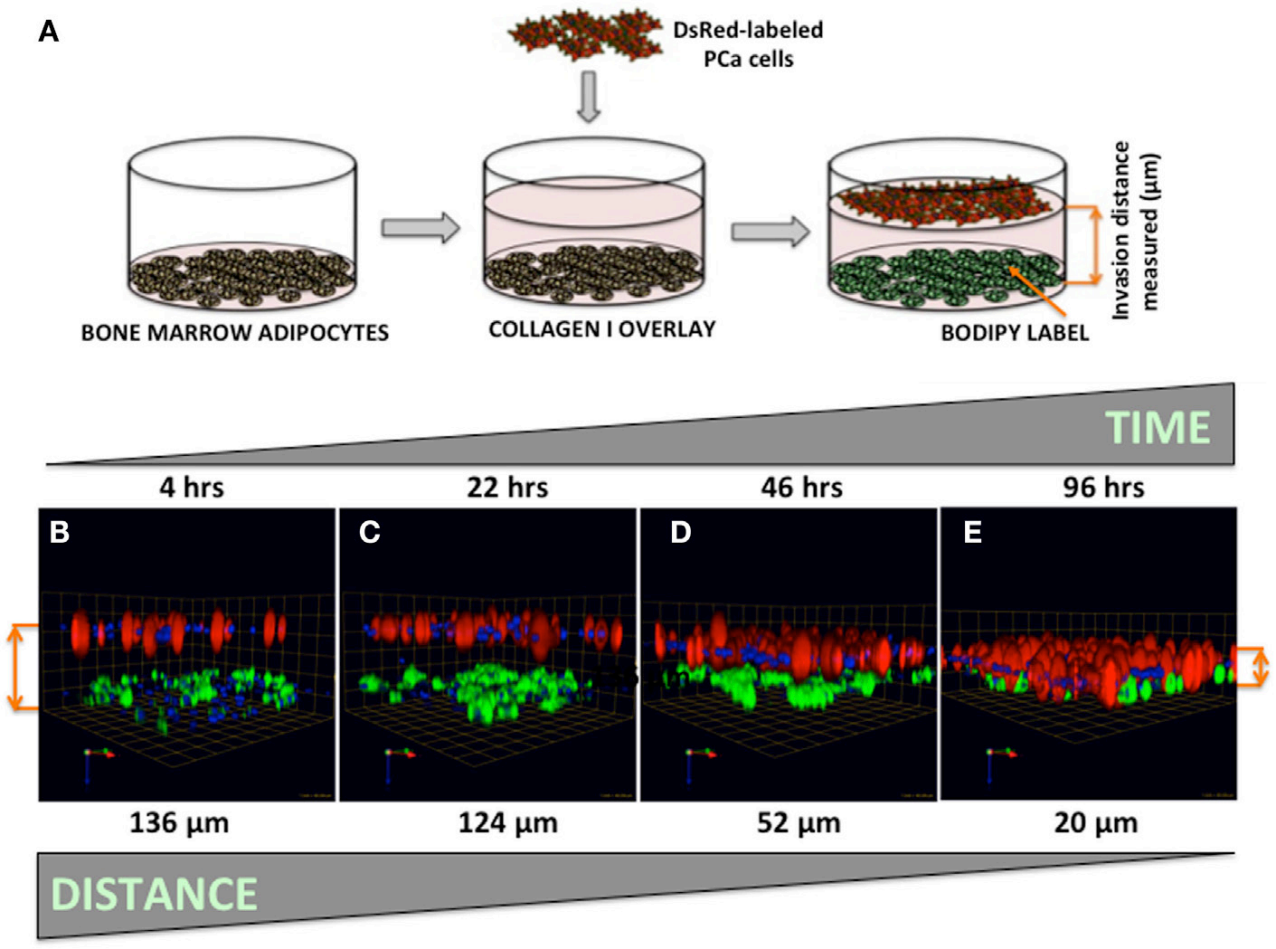
**Three-dimensional invasion of tumor cells toward bone marrow adipocytes**. **(A)** Diagram depicting the experimental setup. Tumor cells invaded through collagen I matrix toward bone marrow adipocytes. Adipocytes (BODIPY, green); Tumor cells (DsRed; red) for 4 h **(B)**, 22 h **(C)**, 46 h **(D)**, and 96 h **(E)** and decreasing distance between the tumor cells and adipocytes over time is shown in micrometers. Green arrow (*X*), red arrow (*Y*), and blue arrow (*Z*) indicate orientation of the spheroid in 3D space.

**Figure 8:**
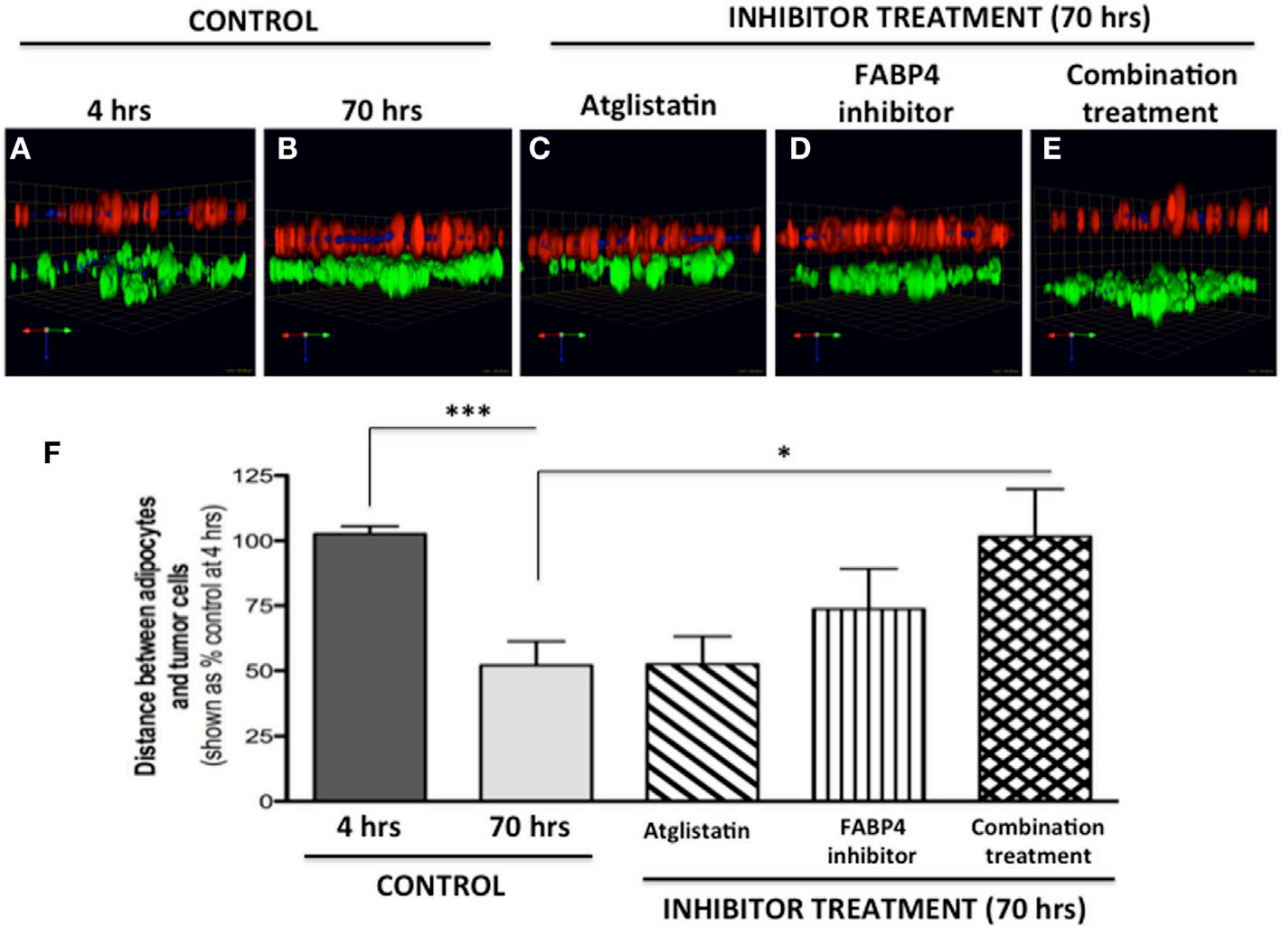
**Inhibition of tumor cell invasion toward adipocytes with FABP4 inhibitor and Atglistatin**. **(A,B)** Invasion under control conditions at 4 h **(A)** and 70 h **(B)**. Adipocytes (BODIPY, green); Tumor cells (DsRed; red). **(C)** Invasion in the presence of 10 μM Atglistatin, **(D)** 1 μM FABP4 inhibitor, **(E)** Invasion in the presence of FABP4 inhibitor and Atglistatin in combination. Green arrow (*X*), red arrow (*Y*), and blue arrow (*Z*) indicate orientation of the spheroid in 3D space **(F)** Quantification of the average distance between PC3 cells and adipocytes under all experimental conditions. Distances between PC3 and adipocyte layer (in micrometers) were measured using the Zeiss LSM Image Browser software. Data were analyzed using GraphPad Prizm and shown as the mean of three independent experiments ± SD (relative to baseline invasion at 4 h). *p < 0.05 and ***p < 0.001 are considered statistically significant.

Bone marrow-derived mBMSC cells were mixed with collagen I matrix and plated in 60-mm dishes (500 μl/dish; ~1,300,000/500 μl). Cells were differentiated into adipocytes over the course of 8–10 days as described above. Post-differentiation, each plate was washed 3× with PBS, with all PBS carefully removed before 1 ml of collagen I was added on top and allowed to polymerize at 37°C for 45 min–1 h. A total of 4 ml of medium, containing 400,000 dsRed-expressing PC3 cells, was gently added on top. Cultures were maintained in a 37°C humidified incubator ventilated with 5% CO_2_ for up to 96 h. At designated time-points, plates were washed with PBS, fixed for 45 min with 3.7% formaldehyde at RT, stained with BODIPY (493/503) (1:1000, 1 h at RT), nuclei were marked with Hoechst dye, and imaged at an extended depth of focus with a Zeiss LSM 510 META NLO confocal microscope using a 20× dipping lens (Figure 7). For inhibitor studies (Figure 8), FABP4 inhibitor (BMS309403; 1 μM) and Atglistatin (10 μM) were added to both the collagen I layer between the adipocytes and the tumor cells as well as the medium, and were replenished in the medium daily.

### Image Reconstruction and Fluorescence Quantification

All images were captured using a Zeiss LSM510 META NLO confocal microscope (Carl Zeiss AG, Göttingen, Germany). For 2D cultures, a 40× oil immersion lens was used, and a single slice was captured. 3D cultures were imaged in 35 mm dishes with a 40× water immersion lens, and *z*-stacks of 2–4 μm optical slices were captured. 3D reconstruction of optical slices was performed using Volocity software (PerkinElmer, Waltham, MA, USA). For quantification of DQ-IV proteolysis, Volocity software was used to determine integrated intensity per image by dividing the total sum of green fluorescence signal (thresholded using intensity) by the number of nuclei in the image. At least three replicates were measured per condition. The totals in each group were averaged and shown as mean ± SEM.

### Measurement and Quantification of Invasion

Invasion cultures in 60 mm dishes were imaged using a 20× water immersion lens, and *z*-stacks of 4 μm increments were captured. 3D reconstruction of optical slices was performed using Volocity software. For quantification of distance traveled by the tumor cells, images were opened in Zeiss LSM Image Browser. After removing the DIC channel, the Ortho tool was used to select and measure the distance from the middle of PC3 (red) layer to the middle of the adipocyte (green) layer. Distances between PC3 and adipocyte layers were recorded from at least three biological triplicates. For inhibitor studies, distances were shown as percent of distance at baseline (4-h control).

### Statistical Analyses

All data analyses were performed using GraphPad Prism Software version 6.05. Data were presented as mean ± SD and statistically analyzed using unpaired Student’s *t*-test. For three or more groups, one-way analysis of variance was used.

## Results

### Three-Dimensional Culture of Prostate Tumor Spheroids in the Absence or Presence of Bone Marrow-Derived Adipocytes

Adipocytes are metabolically active cells that have been shown to promote growth and aggressiveness of several cancers, particularly those that grow in adipocyte-rich microenvironments such as the breast or ovary (10, 35, 36). The fat cells residing specifically in the bone marrow have been increasingly credited with the ability to support and promote metastatic growth in bone (4–9). We have shown previously that exposure of prostate tumor cells to marrow adipocyte-supplied factors increases their proliferation and invasiveness (5). Here, to better mimic physiological conditions and to allow exploration of molecular mechanisms of adipocyte involvement in metastatic tumor growth in bone, we developed a cell culture model that allows 3D growth of prostate cancer cells exposed to adipocytes.

Seeding of the single-cell suspensions of PC3 prostate carcinoma cells on rBM-coated coverslips with 2% rBM overlay results in a formation of tumor spheroids (Figure 1). Cells cultured in their normal growth medium (CONTROL) form compacted, rounded spheroids after 3-day culture (Figure 1B, top panels). In contrast, tumor cells grown in a Transwell coculture with marrow adipocytes (TRANSWELL) form much larger and more disorganized clusters (Figure 1C, top panels). Longer, 5-day culture of PC3 cells results in further growth under either of the conditions, with Transwell cultures exhibiting significantly more robust proliferation and acquiring much more disordered morphology compared with control cultures (Figures 1B,C, bottom panels). Quantification of 3D volumes of the spheroids confirms the growth-promoting effects of adipocytes on tumor cells and indicates potential utility of this experimental system for interrogation of adipocyte–tumor cell interactions (Figure 1D).

### Modeling Metabolic Responses to Marrow Adipocytes

One way that adipocytes can affect tumor cell behavior is by regulating cancer cell metabolism (37). Adipocyte-supplied lipids have been shown to feed into the glycolytic pathway and (38–40) and induce the Warburg effect in tumor cells (41–45). One of the master regulators of metabolic reprograming is hypoxia inducible factor (HIF), a key driver of hypoxic stress (46). Bone marrow adipocytes have a capability of inducing HIF-1α signaling in prostate tumor cells grown in 2D monolayer culture (Podgorski et al., unpublished results^1^). It is, however, well-recognized that tissue dimensionality and associated oxygen status have a potential to profoundly affect hypoxic response in tumor cells (19, 47). Therefore, we examined the utility of our 3D Transwell model to measure hypoxia response to adipocytes. Our 3D cultures established on glass coverslips are easily fixable and suitable for immunocytochemical analyses. We used carbonic anhydrase 9 (CA9), a HIF-1α target gene (48), as a measure of hypoxic response in our system. The immunofluorescence analysis of CA9 protein revealed a significant increase and typical membrane localization in 2D tumor cell cultures exposed to adipocytes (Figures 2A,B), indicating activation of HIF-1α signaling. Interestingly, cells grown in 3D culture showed robust CA9 expression even under control conditions (Figure 2D). However, although tumor cells grown in transwell coculture with adipocytes expectedly formed significantly larger, disorganized structures (Figures 2E,H,I,L,M), there was no visible increase in CA9 expression compared with spheroids grown under control conditions (Figures 2D,F,G,J,K). This was further confirmed by the quantification of CA9 fluorescence/nuclei in each of the 3D structures (data not shown). 3D growth alone appears to activate HIF-1α signaling, which is consistent with the reports of heterogeneous oxygen distribution in 3D cultures (47). This speaks to the importance of considering tissue architecture while examining metabolic responses within the tumor microenvironment. Further studies are underway to validate these findings.

One important consequence of HIF-1α activation is the induction of glycolytic phenotype in tumor cells. We performed immunofluorescence analysis of expression and localization of hexokinase-2 (HK2), a critical enzyme in the first step of glycolysis, that elicits its functions by binding to the voltage-dependent anion channels (VDAC) in the outer mitochondrial membrane (49, 50). Low HK2 expression was detected in monolayer cultures of PC3 cells (Figure 3A), with robust increases in HK2 fluorescence upon exposure to adipocytes (Figure 3E). This is consistent with adipocyte-induced Warburg phenotype in these cells (see footnote text 1). Mitotracker labeling (Figures 3B–D,F–H) showed no observable differences in the number of mitochondria between the control and Transwell conditions. The majority of the HK2 appeared to co-localize with mitochondria (Figures 3C,G), which is consistent with the literature evidence demonstrating that approximately 80% of total HK2 is bound to the mitochondrial VDAC (51).

In stark contrast to the monolayer cultures, a significant HK2 expression was observed in PC3 3D spheroids even under control conditions (Figures 3I,K–N). This indicates a potential enhancement of glycolytic phenotype, consistent with activation of HIF-1α signaling by 3D culture (Figure 2). High HK2 fluorescence closely mirroring mitochondrial pattern was also observed in 3D cultures grown under Transwell conditions (Figures 3J,P–S). However, HK2 fluorescence intensity was not significantly higher than in control cultures, indicating no significant enhancement of HK2 levels by coculture with adipocytes (data not shown).

### Examining Effects of Marrow Adipocyte Presence on Tumor Response to Bone Marrow Macrophages

Bone represents a complex, dynamic microenvironment and establishment of metastatic lesions in the skeleton requires tumor cell interactions with a number of cell types in the marrow. To examine the suitability of our cell culture system to study multi-cell interactions, we incorporated BMMs into 3D tumor spheroids growing under control and Transwell conditions (Figure 4A). Pre-labeling of PC3 tumor cells with CTO allowed visualization of the tumor growing in 3D culture (Figures 4B,C). We tracked the incorporation of BMMs into the tumor spheroid by F4/80 immunofluorescence staining. Tumors growing under Transwell conditions with adipocytes attracted more macrophages than those cultured under control conditions (Figures 4B,C; green fluorescence). Incorporation of BMMs into the spheroid was also visible in the optical slices taken in the middle of the spheroid (Figures 4D–G). These observations suggest that adipocytes might be inducing phenotypic changes in the tumor cells and/or macrophages to promote the BMM affinity for the tumor. Further studies are needed to confirm this hypothesis.

### Imaging Live Tumor Cell-Driven Proteolysis in the Presence of Marrow Adipocytes

To grow and thrive in their surrounding environments, tumors have to interact with the extracellular matrix (ECM) via biochemical and mechanical processes. Proteolysis of the ECM is one of the key aspects of tumor invasion (52, 53), and we have previously shown that exposure to adipocyte-derived factors increases invasive potential of prostate carcinoma cells (5). One way to effectively visualize ECM proteolysis by the tumor cells and other cell types residing in the tumor microenvironment is through a confocal microscopy assay utilizing quenched-fluorescent (DQ) proteins, an approach developed by the Sloane laboratory over a decade ago (53). We have previously utilized this technique to demonstrate that prostate carcinoma cells are capable of degrading quenched fluorescent derivatives of collagen IV (basement membrane) and collagen I (organic matrix of the bone) (33). Therefore, we used this approach to determine whether exposure to adipocyte-derived factors affects the collagen IV degradation in our model. PC3 cells plated on rBM containing DQ-IV were allowed to establish into spheroids by growing for 3 days under control or Transwell conditions before adding CTO-labeled BMMs and allowing cultures to continue for additional 2 days (Figure 5A). Degradation of the DQ-collagen IV, observed as green fluorescence, was captured and quantified at the end of 5-day culture. In agreement with our previous findings (33), fluorescent cleavage products of DQ-collagen IV were found both intracellularly and pericellularly (Figures 5B–E). Similar levels of fluorescence were detected in PC3 cells cultured alone (without BMMs) regardless if they were under control or Transwell conditions (Figures 5B,D,F,H,J), suggesting no measurable contribution of adipocytes to tumor-driven proteolysis.

Since macrophages are important sources of proteases and key contributors to tumor-associated proteolysis (34, 53), we also measured and quantified the DQ-IV fluorescence following the 2-day coculture of pre-established tumor spheroids exposed to macrophages (Figures 5C,E,G,I,J). Surprisingly, despite the significant infiltration of BMMs into tumor spheroids exposed to adipocytes in a Transwell coculture and visible BMM-associated proteolysis (Figures 5E,I), there was no significant increase in overall tumor-associated DQ-IV degradation compared with cultures grown without macrophages (Figure 5J).

Interestingly, when macrophages were incorporated into the culture at the time of seeding the tumor cells (Figure 6A), significant changes were observed in the morphology of the resulting spheroids and in the tumor-associated proteolysis. Specifically, under control conditions, addition of BMMs led to formation of less compacted and more disorganized tumor spheroids compared with tumor cells cultured alone (Figures 6B,F vs. Figures 6C,G). This change in morphology was also associated with a significant increase in DQ-IV proteolysis (Figure 6J). Even more pronounced effects of BMMs were observed in cultures exposed to adipocytes in the Transwell system (Figure 6D,H vs. Figures 6E,I). Formation of highly disorganized tumor cell/BMM clusters with high levels of DQ-IV fluorescence (Figure 6J) suggested potentially important contribution of adipocytes to tumor- and BMM-driven proteolytic activity in this model.

### Quantifying Tumor Cell Invasion Toward Adipocytes in Three-Dimensional System

Our previous work has shown that prostate carcinoma cells exposed to media conditioned by marrow adipocytes take up the fat cell-supplied lipids in a process that results in accelerated tumor growth and invasiveness (5). Prostate tumor cells have been reported to be attracted to adipocyte-rich areas of the bone marrow, where metastases commonly occur (54, 55), and the translocation of adipocyte-stored lipids has been linked to increased tumor cell motility (56). The mechanisms behind the tumor cell attraction to marrow fat cells, however, remain poorly understood. Here, we have designed a simple 3D system in which tumor cell invasion toward adipocytes can be easily visualized, measured, and quantified (Figure 7A). We used collagen I, the predominant component of bone, as the matrix separating BODIPY-labeled bone marrow adipocytes (bottom) and DsRed-expressing PC3 cells (top). Tumor cells invaded through collagen I matrix over the course of 96 h (Figures 7B–E), with complete merging of the two cell types at the 96-h time point.

We next tested two compounds with potential to affect adipocyte–tumor cell interactions: a selective inhibitor of fatty acid-binding protein 4 (FABP4) (BMS309403) (5), and Atglistatin, a selective inhibitor of Adipocyte Triglyceride LIpase (ATGL) (57). We chose to inhibit FABP4 based on our previous studies demonstrating upregulation of this lipid transporter in prostate tumor cells exposed to marrow adipocytes, and the apparent efficacy of BMS309403 in inhibiting prostate tumor cell invasion in the 2D Boyden chamber assay (5). We also used Atglistatin as the means of inhibiting adipocyte-driven lipolysis, which we have shown to be induced by prostate tumor cells (see footnote text 1). As expected, under control conditions, the distance between PC3 cells and marrow adipocytes was significantly reduced after 70 h, compared with the baseline at 4 h (Figures 8A,B). Atglistatin alone did not seem to have any effect on tumor cell invasion toward the adipocytes (Figure 8C). Despite potent inhibition of PC3 invasion in 2D (5), only modest inhibition of tumor invasion was observed with FABP4 inhibitor in a 3D assay (Figure 8D), a result potentially due to differences in matrix and experimental conditions between the two assays. Notably, however, the use of both inhibitors in combination significantly inhibited tumor invasion and restored the distance between the two cell types to the levels measured at 4 h (Figures 8E,F). This suggests a potential link between lipid hydrolysis and transport between adipocytes and tumor cells that needs further investigation.

## Discussion

The functional role of bone marrow adipocytes in the growth and aggressiveness of skeletal tumors is an understudied and not well-understood area of cancer research. There is a great need for *in vitro* 3D models that can address the complexity of the bone tumor microenvironment, yet allow dissection of the mechanisms behind the contribution of specific cell types to the metastatic growth. There has been a growing effort to generate 3D models that would represent a bridge between the 2D monolayer cultures and *in vivo* tumor microenvironment, with the idea of capturing the physiological complexity, multiplicity of cell types, ECM composition, and temporal and spatial distribution of soluble factors in the bone microenvironment (21, 28, 58–61). Many of these models have been key to demonstrating the importance of 3D tumor architecture in mechanistic understanding of basic cancer biology and, especially, in evaluating tumor response to therapy (22, 58, 61). Regretfully, however, none of these models to date have addressed the contribution of fat cells, one of most abundant cell types in the adult bone marrow to growth, phenotype, and behavior of metastatic tumor cells in bone.

The *in vitro* approaches we describe herein provide a simple way of examining interactions between bone marrow adipocytes and metastatic tumor cells in a physiologically relevant manner. Our Transwell system combines 3D culture of bone marrow-derived adipocytes with 3D culture of prostate tumor cells to allow paracrine interactions and sharing of nutrients and secreted factors by the two cell types. We show via immunofluorescence analysis of metabolism-associated proteins that under 3D conditions tumor cells have significantly different metabolic responses to adipocytes than tumor cells grown in a monolayer culture. This underlines the importance of employing 3D culture conditions to mimic physiological interactions between marrow adipocytes and metastatic tumor cells. It is noteworthy that the simple design of our 3D system allows easy manipulation, processing, and analysis of the resulting cultures. Because tumor cell cultures are established on glass coverslips they are immediately available for immunocytochemistry and confocal imaging. These samples can also be recovered from the coverslips and processed for protein and RNA analyses (data not shown), allowing for additional data to support the confocal imaging results. We have also shown the suitability of this system to image proteolysis by living cells. We used DQ Collagen IV as a representative component of the basement membrane, but this approach can be easily modified for use with other DQ-labeled matrices, such as DQ Collagen I [a major protein in the bone (33, 62)], or with activity-based probes (63).

Contribution of other bone marrow-derived cell types is an important consideration in the design of physiologically relevant cell culture models. Our data show that BMMs can be easily incorporated into our coculture system. More importantly, we reveal that macrophages are attracted to tumor spheroids exposed to adipocyte-derived factors, and their coculture with tumor cells results in a more aggressive morphology and an enhanced ECM proteolysis by both cell types. This speaks to the importance of multi-cellular design in modeling the effects of specific cell types on tumor phenotype and behavior. Our system allows the manipulation of the design to vary cell types, numbers, and ECM components. Studies are currently ongoing to examine the effects of direct interaction of marrow macrophages with adipocytes on tumor cell growth and aggressiveness.

Previous studies have indicated that prostate tumor cells are attracted to adipocyte-rich areas in the bone marrow (54, 55). Our 3D invasion assay results confirm this attraction and provide a simple approach to evaluate potential targets involved in this process. We utilized our invasion system to inhibit the lipid transporter FABP4, and the main adipocyte lipase ATGL and revealed that ATGL-driven lipolysis and FABP4-driven lipid transport might be important for tumor cell–adipocyte interaction in the bone marrow microenvironment. This not only opens new avenues for investigation but also speaks to the potential utility of this assay as an initial screen to identify factors behind tumor cell invasiveness toward the marrow adipocytes. Many G protein-coupled receptors (GPRCs), including chemokine receptors and receptors of bioactive lipids, have been implicated in tumor cell growth and invasiveness (64). Given the fact that adipocytes are an abundant source of chemokines and lipids, their role in modulating the invasive capacity of tumor cells via GPCRs could be studied in this system. It is also important to mention that this approach is easily modifiable in terms of matrix type and thickness, time of invasion and cell types used, representing a useful tool to examine the functional effects of marrow adipocytes on tumor cell invasiveness.

We acknowledge that our models have limitations. First, we are utilizing adipocytes differentiated from bone marrow mesenchymal cells, which may not be entirely representative of adipocyte population in bone. Emerging data now show that there might be differences in bone marrow fat depending on localization in the bone (17), and we would not be able to capture these differences with our model. Second, we are utilizing murine bone marrow cells in combination with human tumor cells, which may not be entirely clinically relevant. Species-specific osteotropism is an important factor to be considered in design of physiologically relevant models (65). This would be particularly important when utilizing this model to study the effects of adipocyte-derived cytokines and chemokines on tumor cells. Adipokines, such as hepatocyte growth factor (HGF) or interleukin-6 (IL-6) are known to activate their receptors in a species-specific manner (66, 67). Nevertheless, it is important to mention that murine cells in our system can be easily replaced with human derived mesenchymal bone marrow cells to eliminate this issue.

Our model does not entirely recapitulate the bone tumor microenvironment found in the patient with metastatic disease, but it provides a controllable, testable system for examination of the molecular mechanisms behind adipocyte involvement in tumor progression in bone. We believe that the models described herein provide a good compromise between currently utilized 2D adipocyte-tumor cell cocultures and *in vivo* mouse models, where dissection of the specific contribution of adipocytes is not possible. Further development of these *in vitro* culture systems and combining them with other bone metastasis-focused cell culture models will open new strategies to increase our understanding of the role of marrow adipose tissue in metastatic progression.

## Author Contributions

MH carried out most of the data acquisition and analyses and participated in writing of the manuscript. JD carried out some data acquisition and participated in data analyses and writing of the manuscript. IP wrote the manuscript and participated in some of the data acquisition and analyses.

## Conflict of Interest Statement

The authors declare that the research was conducted in the absence of any commercial or financial relationships that could be construed as a potential conflict of interest.

## Footnotes

^1^Diedrich J, Rajagurubandara E, Herroon MK, Mahapatra G, Huttemann M, Podgorski I (unpublished results).

## References

[1] Berry R Rodeheffer MS Rosen CJ Horowitz MC Adipose tissue residing progenitors (adipocyte lineage progenitors and adipose derived stem cells (ADSC)). Curr Mol Biol Rep (2015) 1(3):101–9. 10.1007/s40610-015-0018-y 26526875 PMC4624461

[2] Georgiou KR Hui SK Xian CJ. Regulatory pathways associated with bone loss and bone marrow adiposity caused by aging, chemotherapy, glucocorticoid therapy and radiotherapy. Am J Stem Cells (2012) 1(3):205–24. 23671809 PMC3636730

[3] Cawthorn WP Scheller EL Learman BS Parlee SD Simon BR Mori H Bone marrow adipose tissue is an endocrine organ that contributes to increased circulating adiponectin during caloric restriction. Cell Metab (2014) 20(2):368–75. 10.1016/j.cmet.2014.06.003 24998914 PMC4126847

[4] Hardaway AL Herroon MK Rajagurubandara E Podgorski I. Marrow adipocyte-derived CXCL1 and CXCL2 contribute to osteolysis in metastatic prostate cancer. Clin Exp Metastasis (2015) 32(4):353–68. 10.1007/s10585-015-9714-5 25802102 PMC4393805

[5] Herroon MK Rajagurubandara E Hardaway AL Powell K Turchick A Feldmann D Bone marrow adipocytes promote tumor growth in bone via FABP4-dependent mechanisms. Oncotarget (2013) 4(11):2108–23. 10.18632/oncotarget.1482 24240026 PMC3875773

[6] Templeton ZS Lie WR Wang W Rosenberg-Hasson Y Alluri RV Tamaresis JS Breast cancer cell colonization of the human bone marrow adipose tissue niche. Neoplasia (2015) 17(12):849–61. 10.1016/j.neo.2015.11.005 26696367 PMC4688564

[7] Caers J Deleu S Belaid Z De Raeve H Van Valckenborgh E De Bruyne E Neighboring adipocytes participate in the bone marrow microenvironment of multiple myeloma cells. Leukemia (2007) 21(7):1580–4. 10.1038/sj.leu.2404658 17377589

[8] Liu Z Xu J He J Liu H Lin P Wan X Mature adipocytes in bone marrow protect myeloma cells against chemotherapy through autophagy activation. Oncotarget (2015) 6(33):34329–41. 10.18632/oncotarget.6020 26455377 PMC4741456

[9] Chen GL Luo Y Eriksson D Meng X Qian C Bäuerle T High fat diet increases melanoma cell growth in the bone marrow by inducing osteopontin and interleukin 6. Oncotarget (2016) 7(18):26653–69. 10.18632/oncotarget.8474 27049717 PMC5042005

[10] Hardaway AL Herroon MK Rajagurubandara E Podgorski I. Bone marrow fat: linking adipocyte-induced inflammation with skeletal metastases. Cancer Metastasis Rev (2014) 33(2–3):527–43. 10.1007/s10555-013-9484-y 24398857 PMC4154371

[11] Morris E Edwards C The role of bone marrow adipocytes in bone metastasis. J Bone Oncol (2016). 10.1016/j.jbo.2016.03.006 PMC5063230 27761371

[12] Picon-Ruiz M Pan C Drews-Elger K Jang K Besser AH Zhao D Interactions between adipocytes and breast cancer cells stimulate cytokine production and drive Src/Sox2/miR-302b-mediated malignant progression. Cancer Res (2016) 76(2):491–504. 10.1158/0008-5472.CAN-15-0927 26744520

[13] Salameh TS Le TT Nichols MB Bauer E Cheng J Camarillo IG. An ex vivo co-culture model system to evaluate stromal-epithelial interactions in breast cancer. Int J Cancer (2013) 132(2):288–96. 10.1002/ijc.27672 22696278

[14] Santander AM Lopez-Ocejo O Casas O Agostini T Sanchez L Lamas-Basulto E Paracrine interactions between adipocytes and tumor cells recruit and modify macrophages to the mammary tumor microenvironment: the role of obesity and inflammation in breast adipose tissue. Cancers (Basel) (2015) 7(1):143–78. 10.3390/cancers7010143 25599228 PMC4381255

[15] Wang C Gao C Meng K Qiao H Wang Y. Human adipocytes stimulate invasion of breast cancer MCF-7 cells by secreting IGFBP-2. PLoS One (2015) 10(3):e0119348. 10.1371/journal.pone.0119348 25747684 PMC4352027

[16] Dirat B Bochet L Dabek M Daviaud D Dauvillier S Majed B Cancer-associated adipocytes exhibit an activated phenotype and contribute to breast cancer invasion. Cancer Res (2011) 71(7):2455–65. 10.1158/0008-5472.CAN-10-3323 21459803

[17] Scheller EL Cawthorn WP Burr AA Horowitz MC MacDougald OA. Marrow adipose tissue: trimming the fat. Trends Endocrinol Metab (2016) 27(6):392–403. 10.1016/j.tem.2016.03.016 27094502 PMC4875855

[18] Pampaloni F Reynaud EG Stelzer EH. The third dimension bridges the gap between cell culture and live tissue. Nat Rev Mol Cell Biol (2007) 8(10):839–45. 10.1038/nrm2236 17684528

[19] Weiswald LB Bellet D Dangles-Marie V. Spherical cancer models in tumor biology. Neoplasia (2015) 17(1):1–15. 10.1016/j.neo.2014.12.004 25622895 PMC4309685

[20] Chambers KF Mosaad EM Russell PJ Clements JA Doran MR 3D cultures of prostate cancer cells cultured in a novel high-throughput culture platform are more resistant to chemotherapeutics compared to cells cultured in monolayer. PLoS One (2014) 9(11):e111029 10.1371/journal.pone.0111029 25380249 PMC4224379

[21] Fong EL Wan X Yang J Morgado M Mikos AG Harrington DA A 3D in vitro model of patient-derived prostate cancer xenograft for controlled interrogation of in vivo tumor-stromal interactions. Biomaterials (2016) 77:164–72. 10.1016/j.biomaterials.2015.10.059 26599623 PMC4684431

[22] Lovitt CJ Shelper TB Avery VM. Advanced cell culture techniques for cancer drug discovery. Biology (Basel) (2014) 3(2):345–67. 10.3390/biology3020345 24887773 PMC4085612

[23] Strand DW Hayward SW. Modeling stromal-epithelial interactions in disease progression. Discov Med (2010) 9(49):504–11. 20587339 PMC4195242

[24] Shamir ER Ewald AJ. Three-dimensional organotypic culture: experimental models of mammalian biology and disease. Nat Rev Mol Cell Biol (2014) 15(10):647–64. 10.1038/nrm3873 25237826 PMC4352326

[25] Nyga A Neves J Stamati K Loizidou M Emberton M Cheema U. The next level of 3D tumour models: immunocompetence. Drug Discov Today (2016). 10.1016/j.drudis.2016.04.010 27113911

[26] Sameni M Anbalagan A Olive MB Moin K Mattingly RR Sloane BF. MAME models for 4D live-cell imaging of tumor: microenvironment interactions that impact malignant progression. J Vis Exp (2012) (60):3661. 10.3791/3661 22371028 PMC3376933

[27] Dovas A Patsialou A Harney AS Condeelis J Cox D. Imaging interactions between macrophages and tumour cells that are involved in metastasis in vivo and in vitro. J Microsc (2013) 251(3):261–9. 10.1111/j.1365-2818.2012.03667.x 23198984 PMC4000025

[28] Salamanna F Contartese D Maglio M Fini M. A systematic review on in vitro 3d bone metastases models. A new horizon to recapitulate the native clinical scenario? Oncotarget (2016). 10.18632/oncotarget.8394 27027241 PMC5190136

[29] Podgorski I Linebaugh BE Koblinski JE Rudy DL Herroon MK Olive MB Bone marrow-derived cathepsin K cleaves SPARC in bone metastasis. Am J Pathol (2009) 175(3):1255–69. 10.2353/ajpath.2009.080906 19700761 PMC2731144

[30] Herroon MK Rajagurubandara E Rudy DL Chalasani A Hardaway AL Podgorski I. Macrophage cathepsin K promotes prostate tumor progression in bone. Oncogene (2013) 32(12):1580–93. 10.1038/onc.2012.166 22614014 PMC3913739

[31] Shaw KR Wrobel CN Brugge JS. Use of three-dimensional basement membrane cultures to model oncogene-induced changes in mammary epithelial morphogenesis. J Mammary Gland Biol Neoplasia (2004) 9(4):297–310. 10.1007/s10911-004-1402-z 15838601 PMC1509102

[32] Jedeszko C Sameni M Olive MB Moin K Sloane BF. Visualizing protease activity in living cells: from two dimensions to four dimensions. Curr Protoc Cell Biol (2008) Chapter 4:Unit4.20. 10.1002/0471143030.cb0420s39 18551423 PMC3782287

[33] Podgorski I Linebaugh BE Sameni M Jedeszko C Bhagat S Cher ML Bone microenvironment modulates expression and activity of cathepsin B in prostate cancer. Neoplasia (2005) 7(3):207–23. 10.1593/neo.04349 15799821 PMC1501133

[34] Sameni M Dosescu J Moin K Sloane BF. Functional imaging of proteolysis: stromal and inflammatory cells increase tumor proteolysis. Mol Imaging (2003) 2(3):159–75. 10.1162/153535003322556903 14649059

[35] Nieman KM Romero IL Van Houten B Lengyel E. Adipose tissue and adipocytes support tumorigenesis and metastasis. Biochim Biophys Acta (2013) 1831(10):1533–41. 10.1016/j.bbalip.2013.02.010 23500888 PMC3742583

[36] Diedrich J Gusky HC Podgorski I. Adipose tissue dysfunction and its effects on tumor metabolism. Horm Mol Biol Clin Investig (2015) 21(1):17–41. 10.1515/hmbci-2014-0045 25781550 PMC4940190

[37] Martinez-Outschoorn UE Sotgia F Lisanti MP. Power surge: supporting cells “fuel” cancer cell mitochondria. Cell Metab (2012) 15(1):4–5. 10.1016/j.cmet.2011.12.011 22225869

[38] Vaughan M The production and release of glycerol by adipose tissue incubated in vitro. J Biol Chem (1962) 237:3354–8. 13996476

[39] Maeda N Funahashi T Shimomura I. Metabolic impact of adipose and hepatic glycerol channels aquaporin 7 and aquaporin 9. Nat Clin Pract Endocrinol Metab (2008) 4(11):627–34. 10.1038/ncpendmet0980 18852723

[40] Langin D. Control of fatty acid and glycerol release in adipose tissue lipolysis. C R Biol (2006) 329(8):598–607. 10.1016/j.crvi.2005.10.008 16860278

[41] Schwartz B Yehuda-Shnaidman E. Putative role of adipose tissue in growth and metabolism of colon cancer cells. Front Oncol (2014) 4:164. 10.3389/fonc.2014.00164 25019059 PMC4071563

[42] Watson DG Tonelli F Alossaimi M Williamson L Chan E Gorshkova I The roles of sphingosine kinases 1 and 2 in regulating the Warburg effect in prostate cancer cells. Cell Signal (2013) 25(4):1011–7. 10.1016/j.cellsig.2013.01.002 23314175 PMC3595369

[43] Tonelli F Alossaimi M Natarajan V Gorshkova I Berdyshev E Bittman R The roles of sphingosine kinase 1 and 2 in regulating the metabolome and survival of prostate cancer cells. Biomolecules (2013) 3(2):316–33. 10.3390/biom3020316 24970170 PMC4030851

[44] Manzi L Costantini L Molinari R Merendino N Effect of dietary omega-3 polyunsaturated fatty acid DHA on glycolytic enzymes and Warburg phenotypes in cancer. Biomed Res Int (2015) 2015:137097 10.1155/2015/137097 26339588 PMC4538308

[45] Baenke F Peck B Miess H Schulze A. Hooked on fat: the role of lipid synthesis in cancer metabolism and tumour development. Dis Model Mech (2013) 6(6):1353–63. 10.1242/dmm.011338 24203995 PMC3820259

[46] Kroemer G Pouyssegur J Tumor cell metabolism: cancer’s Achilles’ heel. Cancer Cell (2008) 13(6):472–82. 10.1016/j.ccr.2008.05.005 18538731

[47] DelNero P Lane M Verbridge SS Kwee B Kermani P Hempstead B 3D culture broadly regulates tumor cell hypoxia response and angiogenesis via pro-inflammatory pathways. Biomaterials (2015) 55:110–8. 10.1016/j.biomaterials.2015.03.035 25934456 PMC4417672

[48] Chiche J Brahimi-Horn MC Pouyssegur J. Tumour hypoxia induces a metabolic shift causing acidosis: a common feature in cancer. J Cell Mol Med (2010) 14(4):771–94. 10.1111/j.1582-4934.2009.00994.x 20015196 PMC3823111

[49] Mathupala SP Ko YH Pedersen PL. Hexokinase II: cancer’s double-edged sword acting as both facilitator and gatekeeper of malignancy when bound to mitochondria. Oncogene (2006) 25(34):4777–86. 10.1038/sj.onc.1209603 16892090 PMC3385868

[50] Pedersen PL Mathupala S Rempel A Geschwind JF Ko YH. Mitochondrial bound type II hexokinase: a key player in the growth and survival of many cancers and an ideal prospect for therapeutic intervention. Biochim Biophys Acta (2002) 1555(1–3):14–20. 10.1016/S0005-2728(02)00248-7 12206885

[51] Arora KK Pedersen PL. Functional significance of mitochondrial bound hexokinase in tumor cell metabolism. Evidence for preferential phosphorylation of glucose by intramitochondrially generated ATP. J Biol Chem (1988) 263(33):17422–8. 3182854

[52] Sahai E. Mechanisms of cancer cell invasion. Curr Opin Genet Dev (2005) 15(1):87–96. 10.1016/j.gde.2004.12.002 15661538

[53] Sloane BF Sameni M Podgorski I Cavallo-Medved D Moin K. Functional imaging of tumor proteolysis. Annu Rev Pharmacol Toxicol (2006) 46:301–15. 10.1146/annurev.pharmtox.45.120403.095853 16402907

[54] Brown MD Hart CA Gazi E Bagley S Clarke NW. Promotion of prostatic metastatic migration towards human bone marrow stoma by Omega 6 and its inhibition by omega 3 PUFAs. Br J Cancer (2006) 94(6):842–53. 10.1038/sj.bjc.6603030 16523199 PMC2361380

[55] Gazi E Gardner P Lockyer NP Hart CA Brown MD Clarke NW. Direct evidence of lipid translocation between adipocytes and prostate cancer cells with imaging FTIR microspectroscopy. J Lipid Res (2007) 48(8):1846–56. 10.1194/jlr.M700131-JLR200 17496269

[56] Brown MD Hart C Gazi E Gardner P Lockyer N Clarke N. Influence of omega-6 PUFA arachidonic acid and bone marrow adipocytes on metastatic spread from prostate cancer. Br J Cancer (2010) 102(2):403–13. 10.1038/sj.bjc.6605481 19997104 PMC2816655

[57] Mayer N Schweiger M Romauch M Grabner GF Eichmann TO Fuchs E Development of small-molecule inhibitors targeting adipose triglyceride lipase. Nat Chem Biol (2013) 9(12):785–7. 10.1038/nchembio.1359 24096302 PMC3829776

[58] Fitzgerald KA Guo J Tierney EG Curtin CM Malhotra M Darcy R The use of collagen-based scaffolds to simulate prostate cancer bone metastases with potential for evaluating delivery of nanoparticulate gene therapeutics. Biomaterials (2015) 66:53–66. 10.1016/j.biomaterials.2015.07.019 26196533

[59] Holen I Nutter F Wilkinson JM Evans CA Avgoustou P Ottewell PD. Human breast cancer bone metastasis in vitro and in vivo: a novel 3D model system for studies of tumour cell-bone cell interactions. Clin Exp Metastasis (2015) 32(7):689–702. 10.1007/s10585-015-9737-y 26231669

[60] Marlow R Dontu G. Modeling the breast cancer bone metastatic niche in complex three-dimensional cocultures. Methods Mol Biol (2015) 1293:213–20. 10.1007/978-1-4939-2519-3_12 26040690

[61] Xu X Farach-Carson MC Jia X Three-dimensional in vitro tumor models for cancer research and drug evaluation. Biotechnol Adv (2014) 32(7):1256–68. 10.1016/j.biotechadv.2014.07.009 25116894 PMC4171250

[62] Herroon MK Sharma R Rajagurubandara E Turro C Kodanko JJ Podgorski I. Photoactivated inhibition of cathepsin K in a 3D tumor model. Biol Chem (2016) 397(6):571–82. 10.1515/hsz-2015-0274 26901495 PMC5901740

[63] Ben-Aderet L Merquiol E Fahham D Kumar A Reich E Ben-Nun Y Detecting cathepsin activity in human osteoarthritis via activity-based probes. Arthritis Res Ther (2015) 17:69. 10.1186/s13075-015-0586-5 25889265 PMC4415352

[64] Dorsam RT Gutkind JS. G-protein-coupled receptors and cancer. Nat Rev Cancer (2007) 7(2):79–94. 10.1038/nrc2069 17251915

[65] Kuperwasser C Dessain S Bierbaum BE Garnet D Sperandio K Gauvin GP A mouse model of human breast cancer metastasis to human bone. Cancer Res (2005) 65(14):6130–8. 10.1158/0008-5472.CAN-04-1408 16024614

[66] Jeffers M Rong S Vande Woude GF. Hepatocyte growth factor/scatter factor-met signaling in tumorigenicity and invasion/metastasis. J Mol Med (Berl) (1996) 74(9):505–13. 10.1007/BF00204976 8892055

[67] Hammacher A Ward LD Weinstock J Treutlein H Yasukawa K Simpson RJ. Structure-function analysis of human IL-6: identification of two distinct regions that are important for receptor binding. Protein Sci (1994) 3(12):2280–93. 10.1002/pro.5560031213 7538847 PMC2142761

